# Weighting Methods for Rare Event Identification From Imbalanced Datasets

**DOI:** 10.3389/fdata.2021.715320

**Published:** 2021-12-23

**Authors:** Jia He, Maggie X. Cheng

**Affiliations:** Department of Applied Mathematics, Illinois Institute of Technology, Chicago, IL, United States

**Keywords:** imbalanced dataset, bias, classification, machine learning, rare event

## Abstract

In machine learning, we often face the situation where the event we are interested in has very few data points buried in a massive amount of data. This is typical in network monitoring, where data are streamed from sensing or measuring units continuously but most data are not for events. With imbalanced datasets, the classifiers tend to be biased in favor of the main class. Rare event detection has received much attention in machine learning, and yet it is still a challenging problem. In this paper, we propose a remedy for the standing problem. Weighting and sampling are two fundamental approaches to address the problem. We focus on the weighting method in this paper. We first propose a boosting-style algorithm to compute class weights, which is proved to have excellent theoretical property. Then we propose an adaptive algorithm, which is suitable for real-time applications. The adaptive nature of the two algorithms allows a controlled tradeoff between true positive rate and false positive rate and avoids excessive weight on the rare class, which leads to poor performance on the main class. Experiments on power grid data and some public datasets show that the proposed algorithms outperform the existing weighting and boosting methods, and that their superiority is more noticeable with noisy data.

## 1 Introduction

In this paper, we study the problem of learning with an imbalanced dataset. In classification, this is also called rare events problem, in which there are thousands of times fewer *yes* cases than *no* cases. The yes cases are called events. Usually the events are what we are interested in, which may have very few occurrences while the nonevent cases are abundant. This is typical in network monitoring applications, where data representing events are only a tiny portion of the entire dataset. For instance, we may have a fault or anomaly observed in 1 month’s worth of data while all other observations are nonevents. Using machine learning approach forevent detection and identification would require training a machine learning algorithm with these data, but the scarce representation of events in the dataset makes learning the rare event difficult.

It has been reported in the statistics literature that rare events are difficult to predict [see King and Zeng (2001) and others]. In Weiss (2004), it is pointed out that with imbalanced datasets, the learning algorithms are biased in favor of the class priors. The statistical procedure to predict the event, such as Logistic Regression, often underestimates the probability of the rare events. Such procedures will have a high overall prediction accuracy mainly due to the correct predictions on the large number of nonevent cases, but the *recall* metrics, defined as the fraction of true positives that have been successfully predicted, is extremely low. Such performance does not serve the purpose of event detection, since what we are interested in is the event. For event detection in a networked system, having a missed detection on important events has more detrimental effect than having a false alarm. Oftentimes we are willing to improve the detection rate even if it will generate more false positives. However, using the standard classifiers, the cost associated with misclassification on either class has the same contribution in the cost function.

To address the problem, we need to give more importance to the rare class in the cost function. This can be done either by directly changing the number of examples in each class in the training set, i.e., by sampling (Cahyana et al., 2019), or by changing the weights of the classes, which also changes the distribution of the classes. A review article (Li and Mao, 2014) provided an in-depth study of data sampling strategies and weight-modification strategies from the Bayesian classification point of view. In addition, the number of classes is also not limited to two classes. It can be extended to multiple-classes (Tanha et al., 2020).

Sampling has the merit of simplicity but it also has limitations. First, there are two forms of rarity as pointed out in Weiss (2004): absolute rarity and relative rarity. While relative rarity can be corrected by under-sampling the main class, absolute rarity can only resort to oversampling the rare class. However there is an issue with this approach—in case there are outliers in the data, the oversampled outliers will ruin the prediction performance. Moreover, under-sampling may also cause loss of information. It is necessary to consider an alternative way to address the rarity issue.

In this paper, we focus on weighting methods. Unlike the previous weighting methods that use the population information or the relative rarity in the sample to decide the weights, we develop algorithms to compute the weights during the training process. The weighting algorithms can work with any classifier. In this paper, we use Logistic Regression, Random Forest, and Support Vector Machine to demonstrate its effectiveness. The proposed algorithms have the advantages of not relying on unknown population information, and being able to improve the prediction performance of the rare class with a controllable tradeoff with the main class. Most importantly, this is the best approach to deal with absolute rarity, which poses great challenges to other methods.

In practice, a user does not have to choose between a weighting method and a sampling method. An ensemble approach that combines sampling techniques and weighting techniques can achieve the best of the two worlds. For instance, (Guo and Viktor, 2004) combines generating synthetic data and boosting procedures to improve the predictive accuracies of both the majority and minority classes. An improvement on the weighing method also contributes to the ensemble techniques.

The rest of the paper is organized as follows: in Section 2, we cover the preliminaries for classification with imbalanced dataset; in Section 3, we propose two weighting algorithms, DiffBoost, and AdaClassWeight. They can address both forms of rarity by adaptively adding weights and combining weighting with boosting; and subsequently in Section 4, we explain how to train a classifier under the computed weights; in Section 5, we provide performance results for the proposed algorithm, along with comparison with related methods; and in Section 6, we conclude the paper with outlook for future work.

## 2 Classification With Imbalanced Datasets

Among many others (Japkowicz et al., 1995; Liu et al., 2006; Ertekin et al., 2007), using weights is a fundamental approach to address the data imbalance problem in classification. We focus on the use of class weights in this paper, in which we add class weights to the loss function, making it more expensive to have a classification error in the rare class. This is done by assigning the rare class a larger weight and the main class a smaller weight. Weighting is also considered a type of cost-sensitive learning method (Pazzani et al., 1994). In cost-sensitive learning, the cost associated with misclassifying a rare class outweighs the cost of correctly classifying the main class. In King and Zeng (2001), weights are decided based on sample distribution in the population: the rare class weight $w^{+}=\tau /\bar{y}$ and the main class weight $w^{-}=\left(1-\tau \right)/\left(1-\bar{y}\right)$ , where *τ* is the fraction of the rare class in the population, and $\bar{y}$ is the fraction in the sample, respectively. In some applications, population information may be straightforward to know, such as in political activities (King and Zeng, 2001). However, in most other applications, we do not know the class distribution in the population. For convenience, many resort to using the sample information, i.e., in the training set if there are *N* ^+^ examples in the rare class, and *N* ^−^ examples in the main class, the weight would be *N* ^−^/*N* ^+^ for the rare class and 1 for the main class. This method, as we will see later in this paper, has the disadvantage of not considering the absolute rarity, and also not having control over the trade off between the false positive rate and the false negative rate. In case we need to prioritize the rare class, we cannot improve the performance on the rare class further since the fixed weights only reflect the ratio of the examples in the sample.

Similar to the class-weighted methods, there are previous work that use individual weights in the classification algorithms. We briefly review some existing work that are developed based on the idea of introducing a cost for each individual example. The weight update rule was first introduced in AdaBoost (Freund and Schapire, 1995) to force the classifier to be biased towards the minority class.

Given the number of iterations *T*, and training data $\left\{\left(x_{i},y_{i}\right),i=1,\ldots ,N\right\}$ , the AdaBoost algorithm computes the sample weight distribution *D*_*t*_ at the *t*-th iteration. *h*_*t*_ is the classifier trained under weight distribution *D*_*t*_. The weight update rule in AdaBoost is given by

$$D_{t+1}\left(i\right)=\frac{D_{t}\left(i\right)\exp\left(-\alpha_{t}h_{t}\left(x_{i}\right)y_{i}\right)}{Z_{t}},$$ (1)

where *α*_*t*_ is a weight update parameter that needs to be computed in each iteration. *Z*_*t*_ is normalization factor defined as

$$Z_{t}=\underset{i}{\sum }D_{t}\left(i\right)\exp\left(-\alpha_{t}h_{t}\left(x_{i}\right)y_{i}\right).$$ (2)

Based on the weight update rule of AdaBoost, later works AdaC1, AdaC2, and AdaC3 from Sun et al. (2007), CSB1 and CSB2 from Ting (2000), and Adacost from Fan et al. (1999) were developed by associating a cost *C*_*i*_ ≥ 0 with individual examples in Eq. 1. Examples from the minority class are associated with larger costs than those from the majority class.

• AdaC1 modifies Eq. 1 by introducing *C*_*i*_ inside the exponent,

$$D_{t+1}\left(i\right)=\frac{D_{t}\left(i\right)\exp\left(-\alpha_{t}C_{i}h_{t}\left(x_{i}\right)y_{i}\right)}{Z_{t}}.$$ (3)

• AdaC2 adds a cost *C*_*i*_ outside the exponent of Eq. 1,

$$D_{t+1}\left(i\right)=\frac{C_{i}D_{t}\left(i\right)\exp\left(-\alpha_{t}h_{t}\left(x_{i}\right)y_{i}\right)}{Z_{t}}.$$ (4)

• AdaC3 can be considered as a combination of AdaC1 and AdaC2, in which *C*_*i*_ is included both inside and outside the exponent,

$$D_{t+1}\left(i\right)=\frac{C_{i}D_{t}\left(i\right)\exp\left(-\alpha_{t}C_{i}h_{t}\left(x_{i}\right)y_{i}\right)}{Z_{t}}.$$ (5)

• AdaCost also uses a cost inside the exponent of Eq. 1, however, instead of directly using cost item *C*_*i*_, it defines a cost adjustment function $\Gamma_{1_{\left(h_{t}\left(x_{i}\right),y_{i}\right)}}$ based on *C*_*i*_,

$$D_{t+1}\left(i\right)=\frac{D_{t}\left(i\right)\exp\left(-\alpha_{t}\Gamma_{1_{\left(h_{t}\left(x_{i}\right),y_{i}\right)}}h_{t}\left(x_{i}\right)y_{i}\right)}{Z_{t}},$$ (6)

where $\Gamma_{1_{\left(h_{t}\left(x_{i}\right),y_{i}\right)}}$ can be set as Γ_+_ = − 0.5*C*_*i*_ + 0.5 if classified correctly, and Γ_−_ = 0.5*C*_*i*_ + 0.5 otherwise.

All the aforementioned methods involve using a cost. A common drawback of them is that one must manually determine the “optimal” cost, which is predetermined. Arbitrarily selected costs can result in poor classification performance as shown in Section 5. However, there is no better algorithm than exhaustive search to decide the costs. This motivates an algorithm that computes the weights solely from the data and does not depend on any hyper-parameter.

## 3 The Proposed Weighting Methods

Throughout this paper, we assume the rare class is the positive class. Let *N* ^+^ be the number of examples in the rare class, and *N* ^−^ the number of examples in the main class, and *N* ^+^ ≪ *N* ^−^. The class weights are denoted as *w* ^+^ for the rare class and *w* ^−^ for the main class.

Through numerous tests, it is observed that what makes it difficult to learn from an imbalanced dataset is not the relative ratio of the rare class to the main class, rather it is the small number of examples in the rare class. In other words, the absolute rarity matters much more than the relative rarity. This is especially true for Logistic Regression, as the logistic model is often estimated by using maximum likelihood estimation and inherently has the “small-sample bias” issue (King and Zeng, 2001). The simple ratio-based algorithm that uses *w* ^+^/*w* ^−^ = *N* ^−^/*N* ^+^ will only use the ratio information regardless of the sample size and is deemed unable to find the optimal weights. For a dataset with (*N* ^+^, *N* ^−^) = (200, 2000), a weight ratio of *w* ^+^/*w* ^−^ = 10 gives too much weight to the rare class, leading to overfitting the rare class; and for a dataset with (*N* ^+^, *N* ^−^) = (2, 10), using a weight ratio of *w* ^+^/*w* ^−^ = 5 is not enough.

We use two experiments on the Spam data to demonstrate the effect of the absolute rarity. In the first experiment, the training set has a total of 2,200 examples, with $\left(N^{+},N^{-}\right)=\left(200,2000\right)$ . We compare results using a sequence of weights: *w* ^+^/*w* ^−^ ∈ {2, 5, 7.5, 10}. Table 1 shows the results. It is noted that for the simple ratio-based algorithm using *w* ^+^/*w* ^−^ = 10, while the recall is the highest, the precision is the lowest for both training and test, and the sum of recall and precision is the lowest. Therefore, the results support the claim that *w* ^+^/*w* ^−^ = 10 leads to overfitting on the rare class. On the other hand, the algorithm with *w* ^+^/*w* ^−^ = 5 has competitive performance, with a close-to-the-highest recall and the highest sum of recall and precision, indicating that *w* ^+^/*w* ^−^ = 5 is the best among the four options.

**TABLE 1:** Spam Data. The number of examples in the training set is $\left(N^{+},N^{-}\right)=\left(200,2000\right)$ .

	Training		Test	
	Recall	Precision	Recall	Precision
*w* ^+^/*w* ^−^ = 2	0.819	0.804	0.776	0.752
*w* ^+^/*w* ^−^ = 5	0.910	0.716	0.870	0.676
*w* ^+^/*w* ^−^ = 7.5	0.92	0.668	0.887	0.627
*w* ^+^/*w* ^−^ = 10	0.924	0.491	0.897	0.440

For the second experiment, the training set has a total of 12 examples, with $\left(N^{+},N^{-}\right)=\left(2,10\right)$ . We used a sequence of weights: *w* ^+^/*w* ^−^ ∈ {5, 7.5, 10, 12}, and the results are shown in Table 2. It is noted that simply setting *w* ^+^/*w* ^−^ = 5 according to the ratio of the examples in the training set is not enough to have a high recall. The algorithm with *w* ^+^/*w* ^−^ = 12 has the highest recall, and the algorithm with *w* ^+^/*w* ^−^ = 10 has a close-to-the-highest recall and the highest sum for recall and precision.

**TABLE 2:** Spam Data. The number of examples in the training set is $\left(N^{+},N^{-}\right)=\left(2,10\right)$ .

	Training		Test	
	Recall	Precision	Recall	Precision
*w* ^+^/*w* ^−^ = 5	0.800	0.559	0.580	0.239
*w* ^+^/*w* ^−^ = 7.5	0.950	0.388	0.620	0.223
*w* ^+^/*w* ^−^ = 10	1.000	0.382	0.640	0.206
*w* ^+^/*w* ^−^ = 12	1.000	0.382	0.660	0.183

The experiments verified that it is the number of examples in the rare class that determines the error. Therefore, it is necessary to look beyond the ratio and develop an algorithm to find the class weights. We present two algorithms, Differentiated Boosting (DiffBoost) and Adaptive Class Weights (AdaClassWeight).

### 3.1 A Boosting Style Weighting Method: DiffBoost

We define two index sets *I* ^+^ = {*i*: *y*_*i*_ = 1}, and *I* ^−^ = {*i*: *y*_*i*_ = − 1}. We use + to denote the rare class and − to denote the main class. In the algorithm DiffBoost, we compute the weights iteratively under an overarching boosting framework.

Algorithm 1 DiffBoost.

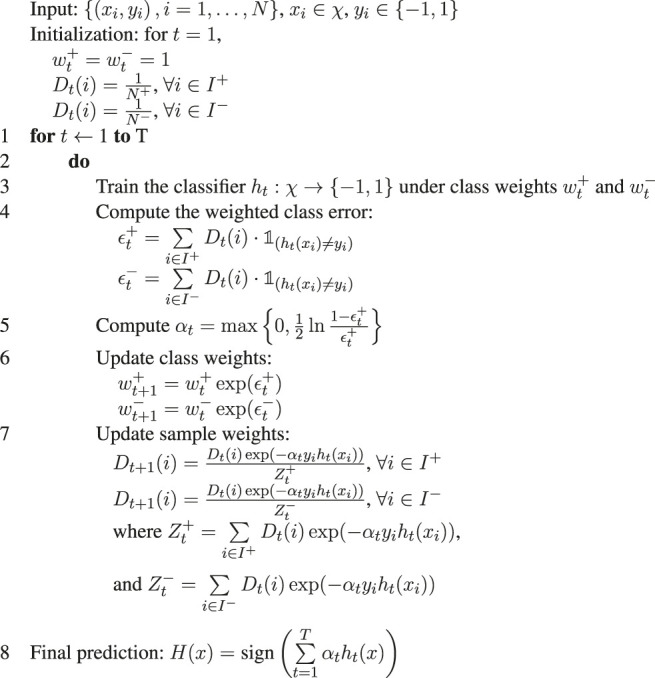

The classifier *h*_*t*_ maps an element in the feature space *χ* to a label. $Z_{t}^{+}$ and $Z_{t}^{-}$ are chosen such that $\underset{i\in I^{+}}{\sum }D_{t+1}\left(i\right)=1$ , and $\underset{i\in I^{-}}{\sum }D_{t+1}\left(i\right)=1$ , and therefore *D*_*t*+1_(*i*), *i* ∈ *I* ^+^ will be a distribution, and *D*_*t*+1_(*i*), *i* ∈ *I* ^−^ will be a distribution. This is the invariant of the algorithm, as it holds from initialization until the algorithm terminates.

Although the algorithm seems to operate symmetrically on both the rare class and the main class, it actually boosts the performance of the rare class more than the main class. This is due to the fact that *N* ^+^ ≪ *N* ^−^, and therefore *D*_*t*_(*i*) takes larger values for *i* ∈ *I* ^+^ than for *i* ∈ *I* ^−^. Initially, the main class is doing well due to the large number of examples, so we have ϵ+ > ϵ− after the first iteration, and then *w* ^+^ becomes larger than *w* ^−^. The algorithm starts to behave in favor of the rare class.

This boosting algorithm has the property that as the iteration number *T* increases, the training error for the rare class monotonically decreases. We outline the proof in the following. The training error of the rare class is given by $\frac{1}{N^{+}}\underset{i\in I^{+}}{\sum }1_{\left(H\left(x_{i}\right)\neq y_{i}\right)}$ .

Theorem 1
$\frac{1}{N^{+}}\underset{i\in I^{+}}{\sum }1_{\left(H\left(x_{i}\right)\neq y_{i}\right)}$ *asymptotically converges to zero as* *T* → *∞* *.*

The proof of Theorem 1 is straightforward from Lemma 1 and Lemma 2.

Lemma 1 *The training error of the rare class has the following bound:*

$$\frac{1}{N^{+}}\underset{i\in I^{+}}{\sum }1_{\left(H\left(x_{i}\right)\neq y_{i}\right)}\leq \overset{T}{\underset{t=1}{\prod }}Z_{t}^{+}.$$

Proof Let $f\left(x\right)=\overset{T}{\underset{t=1}{\sum }}\alpha_{t}h_{t}\left(x\right)$ , i.e., the final prediction *H*(*x*) = sign(*f*(*x*)). From the update rule of *D*_*t*+1_(*i*), and using telescoping, we have that *∀i* ∈ *I* ^+^,

$$\begin{array}{cc} & D_{T+1}\left(i\right)\\ & =D_{T}\left(i\right)\cdot \frac{\exp\left(-\alpha_{T}y_{i}h_{T}\left(x_{i}\right)\right)}{Z_{T}^{+}}\\ & =D_{1}\left(i\right)\cdot \frac{\exp\left(-\alpha_{1}y_{i}h_{1}\left(x_{i}\right)\right)}{Z_{1}^{+}}\ldots \frac{\exp\left(-\alpha_{T}y_{i}h_{T}\left(x_{i}\right)\right)}{Z_{T}^{+}}\\ & =\frac{1}{N^{+}}\cdot \frac{\exp\left(-y_{i}\overset{T}{\underset{t=1}{\sum }}\alpha_{t}h_{t}\left(x_{i}\right)\right)}{\prod_{t=1}^{T}Z_{t}^{+}}\\ & =\frac{1}{N^{+}}\cdot \frac{\exp\left(-y_{i}f\left(x_{i}\right)\right)}{\prod_{t=1}^{T}Z_{t}^{+}} \end{array}$$

Next, we show that the training error of the rare class is bounded from above by $\prod_{t=1}^{T}Z_{t}^{+}$ .

Recall that *H*(*x*_*i*_) = sign(*f*(*x*_*i*_)).

• If *y*_*i*_ and *f*(*x*_*i*_) have the same sign, then $1_{\left(H\left(x_{i}\right)\neq y_{i}\right)}=0$ , and 0 < exp(−*y*_*i*_ *f*(*x*_*i*_)) < 1, thus $1_{\left(H\left(x_{i}\right)\neq y_{i}\right)}\leq \exp\left(-y_{i}f\left(x_{i}\right)\right)$ .
• If *y*_*i*_ and *f*(*x*_*i*_) have different signs, $1_{\left(H\left(x_{i}\right)\neq y_{i}\right)}=1$ , and exp(−*y*_*i*_ *f*(*x*_*i*_)) > 1, thus $1_{\left(H\left(x_{i}\right)\neq y_{i}\right)}\leq \exp\left(-y_{i}f\left(x_{i}\right)\right)$ still holds.

Combining both cases, we have

$$\begin{array}{cc} \frac{1}{N^{+}}\underset{i\in I^{+}}{\sum }1_{\left(H\left(x_{i}\right)\neq y_{i}\right)} & \leq \frac{1}{N^{+}}\underset{i\in I^{+}}{\sum }\exp\left(-y_{i}f\left(x_{i}\right)\right)\\ & =\underset{i\in I^{+}}{\sum }D_{T+1}\left(i\right)\overset{T}{\underset{t=1}{\prod }}Z_{t}^{+}\\ & \overset{\left(a\right)}{=}\overset{T}{\underset{t=1}{\prod }}Z_{t}^{+} \end{array}$$

(a) is due to that $\underset{i\in I^{+}}{\sum }D_{T+1}\left(i\right)=1$ , which is the invariant of the algorithm.

Lemma 2
$\prod_{t=1}^{T}Z_{t}^{+}$ *decreases monotonically as the iteration number* *T* *increases.*

Proof
$$\begin{array}{cc} Z_{t}^{+} & =\underset{i\in I^{+}}{\sum }D_{t}\left(i\right)\exp\left(-\alpha_{t}y_{i}h_{t}\left(x_{i}\right)\right)\\ & =\underset{i\in I^{+},h_{t}\left(x_{i}\right)=y_{i}}{\sum }D_{t}\left(i\right)\exp\left(-\alpha_{t}y_{i}h_{t}\left(x_{i}\right)\right)+\underset{i\in I^{+},h_{t}\left(x_{i}\right)\neq y_{i}}{\sum }D_{t}\left(i\right)\exp\left(-\alpha_{t}y_{i}h_{t}\left(x_{i}\right)\right)\\ & =e^{-\alpha_{t}}\underset{i\in I^{+},h_{t}\left(x_{i}\right)=y_{i}}{\sum }D_{t}\left(i\right)+e^{\alpha_{t}}\underset{i\in I^{+},h_{t}\left(x_{i}\right)\neq y_{i}}{\sum }D_{t}\left(i\right)\\ & =e^{-\alpha_{t}}\left(1-\epsilon_{t}^{+}\right)+e^{\alpha_{t}}\epsilon_{t}^{+} \end{array}$$

Plugging in $\alpha_{t}=\max\left\{0,\frac{1}{2}\ln\frac{1-\epsilon_{t}^{+}}{\epsilon_{t}^{+}}\right\}$ , we have the following,

• When $\epsilon_{t}^{+}<\frac{1}{2}$ , $\alpha_{t}=\frac{1}{2}\ln\frac{1-\epsilon_{t}^{+}}{\epsilon_{t}^{+}}$ ,

$Z_{t}^{+}=\sqrt{\frac{\epsilon_{t}^{+}}{1-\epsilon_{t}^{+}}}\;\left(1-\epsilon_{t}^{+}\right)+\sqrt{\frac{1-\epsilon_{t}^{+}}{\epsilon_{t}^{+}}}\;\epsilon_{t}^{+}<1$ .

• When $\epsilon_{t}^{+}\geq \frac{1}{2}$ , *α*_*t*_ = 0 (i.e., this iteration has no contribution to the final prediction), and $Z_{t}^{+}=1$ .

Therefore $Z_{t}^{+}\leq 1,\forall t$ . Thus, $\prod_{t=1}^{T}Z_{t}^{+}$ decreases monotonically with *T*.

The following analysis shows that $Z_{t}^{+}=1$ is only a transient state and the algorithm will quickly pass this state and enter into an exponential decrease state.

• The applicable scenario for the weighting algorithm is when data is extremely imbalanced and the rare class has very few data points. Under this condition, it is reasonable to assume that the initial weighted class error $\epsilon_{t}^{+}>\epsilon_{t}^{-}$ . During the iteration, $\epsilon_{t}^{+}$ will decrease, and we can stop the iteration if $\epsilon_{t}^{+}<\epsilon_{t}^{-}$ has been achieved in less than *T* iterations.
• When $\epsilon^{+}\geq \frac{1}{2}$ , *α*_*t*_ = 0, *D*_*t*+1_(*i*) = *D*_*t*_(*i*) is unchanged, $w_{t+1}^{+}$ and $w_{t+1}^{-}$ both increase, however $w_{t+1}^{+}$ increases faster since $\epsilon_{t}^{+}>\epsilon_{t}^{-}$ . Therefore, in the next iterations $\epsilon_{t+1}^{+}$ will be decreasing. It continues to decrease until eventually $\epsilon_{t}^{+}$ becomes less than $\frac{1}{2}$ , so that the upper bound starts to decrease again.
• When $\epsilon^{+}<\frac{1}{2}$ , $Z_{t}^{+}=2\sqrt{\epsilon_{t}^{+}\left(1-\epsilon_{t}^{+}\right)}$ . Let $\gamma_{t}=\frac{1}{2}-\epsilon_{t}^{+}$ , we have $Z_{t}^{+}=\sqrt{1-4\gamma_{t}^{2}}\leq e^{-2\gamma_{t}^{2}}$ , and $\prod_{t}Z_{t}^{+}\leq e^{-2\underset{t}{\sum }\gamma_{t}^{2}}$ . In this case, the upper bound decreases exponentially.

Since the upper bound of $\frac{1}{N^{+}}\underset{i\in I^{+}}{\sum }1_{\left(H\left(x_{i}\right)\neq y_{i}\right)}$ decreases monotonically with *T*, and $Z_{t}^{+}=1$ is a transient state and will eventually transform to $Z_{t}^{+}<1$ , we conclude that the training error of the rare class asymptotically converges to zero as *T* → *∞*.

**FIGURE 1:**
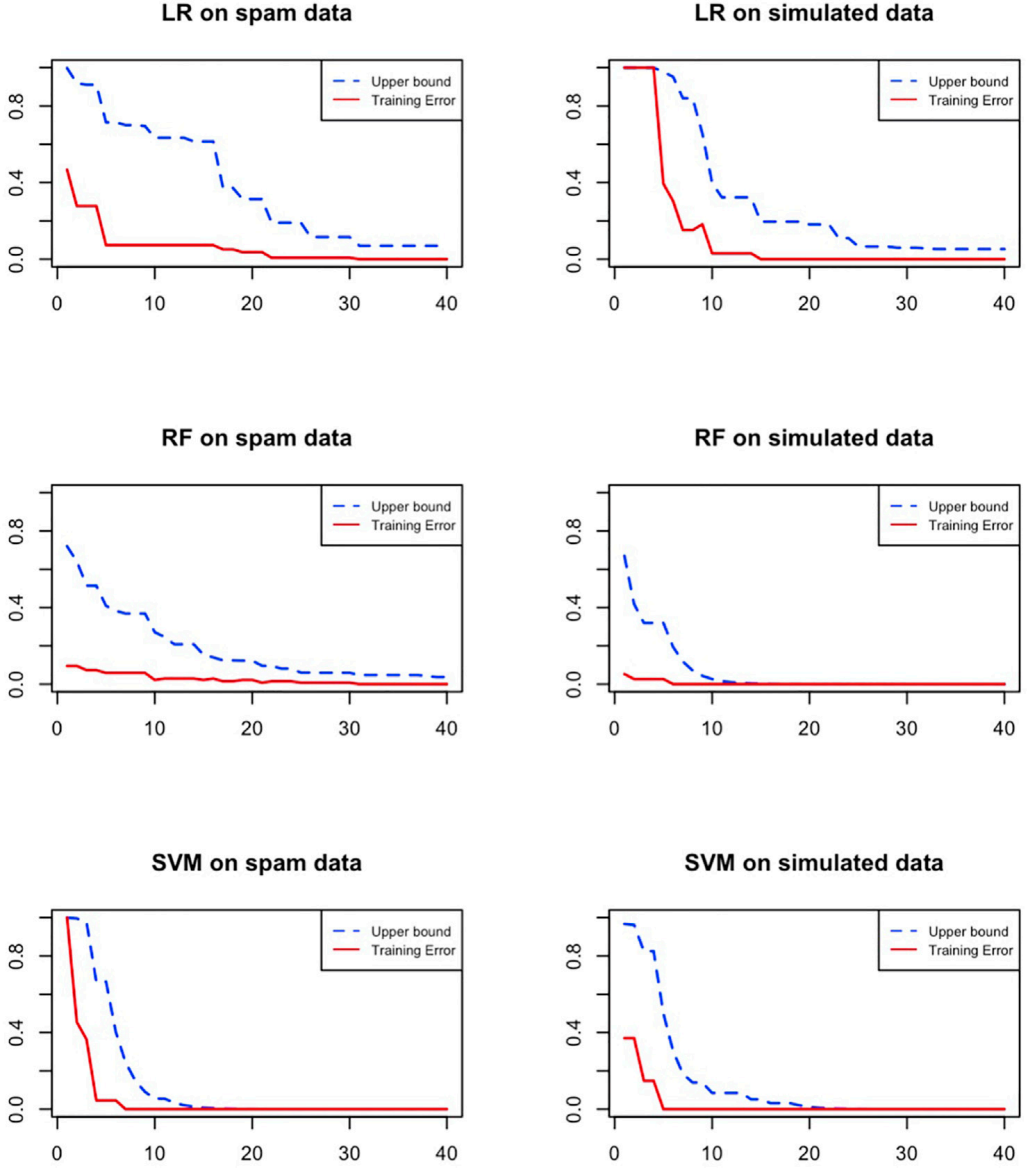
The convergence of the DiffBoost algorithm. Training error of the rare class asymptotically converges to zero as its upper bound decreases monotonically.

Figure 1 shows the upper bound of the training error and the actual training error vs the iteration number by using DiffBoost. Three algorithms, Logistic Regression (LR), Random Forest (RF) and Support Vector Machine (SVM) are tested on two datasets. The Spam dataset is from UCI machine learning repository (Dua and Graff, 2017). The simulated data are generated using a function. We generate a total of 1,832 examples with three predictors *X*_1_, *X*_2_ and *X*_3_. Each predictor variable follows a Gaussian distribution, *X*_1_ ∼ *N*(0, 1), *X*_2_ ∼ *N*(1, 2) and *X*_3_ ∼ *N*(−2, 1.5). The target function is $f\left(X_{1},X_{2},X_{3}\right)=X_{1}+X_{2}+X_{3}-5\left(X_{1}^{2}+X_{2}^{2}+X_{3}^{2}\right)+X_{1}^{3}+X_{2}^{3}+X_{3}^{3}$ . The response variable *Y* = *f*(*X*_1_, *X*_2_, *X*_3_) + *e*, with error term *e* ∼ *N*(0, 3). Binary labels are assigned as *y*_*i*_ = 1 if *y*_*i*_ ≥ 0 and *y*_*i*_ = − 1 otherwise.

The theory and simulation both show that DiffBoost has excellent converging property. However, it still has one issue—ittakes as many iterations to predict a new response as it takes to train the classifier. This problem is inherent to the boosting style algorithms. AdaBoost (Freund and Schapire, 1997) has the same issue. The parameters learned from each iteration as well as *α*_*t*_ must be saved, as the final prediction $H\left(x\right)=\mathrm{sign}\left(\overset{T}{\underset{t=1}{\sum }}\alpha_{t}h_{t}\left(x\right)\right)$ requires *α*_*t*_ and all the parameters used by *h*_*t*_(*x*) for *t* = 1, …, *T*. In the next section, we propose an algorithm that takes only one shot to predict. Training may take many iterations, and can take place off-line, but once we have learned the class weights, prediction takes only one shot. This algorithm will be suitable for real-time applications.

### 3.2 Adaptively Computing Class Weights

Algorithm 2 AdaClassWeight.

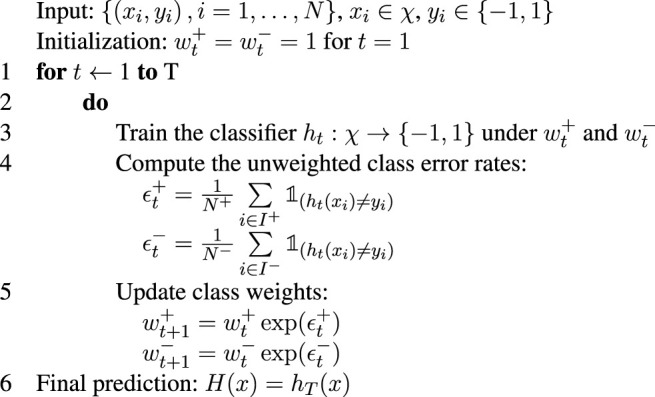

The algorithm, called AdaClassWeight, starts with an unweighted classifier and adaptively increases the weight of the rare class until a stopping criterion is met. Unlike the DiffBoost algorithm, the AdaClassWeight algorithm only uses the parameters of the classifier learned in the final iteration to make a new prediction. DiffBoost would require all the learned parameters in the past *T* iterations in order to make a new prediction. AdaClassWeight also differs from DiffBoost in the way that the class error rates $\epsilon_{t}^{+}$ and $\epsilon_{t}^{-}$ are updated. DiffBoost uses the weighted class error rates while AdaClassWeight uses the unweighted class error rates. If there is misclassification within a class, the unweighted class error rate satisfies 0 < ϵ_*t*_ ≤ 1, so the class weight will increase in the next iteration. However, the class with a larger error rate will increase more, thus to get better classification results in the next iteration.

Remark For the implementation of AdaClassWeight and DiffBoost, we can inject a stopping criterion during the iteration to avoid unnecessary large number of iterations. We can stop whenever the desired error rate for the rare class has been achieved, i.e., we can stop when $\epsilon_{t}^{+}<\epsilon_{t}^{-}$ , or when an absolute error threshold has reached, e.g., $\epsilon_{t}^{+}<0.001$ .

## 4 Incorporating Class Weights Into Classification Algorithms

We show how the class weights *w* ^+^ and *w* ^−^ computed from DiffBoost and AdaClassWeight are used with Logistic Regression, Random Forest, and Support Vector Machine.

### 4.1 Weighted Logistic Regression

Logistic Regression models are usually fit by the maximum likelihood method (Cox, 1958; Hastie et al., 2009). The log-likelihood for *N* observations is given by:

$$L\left(\beta \right)=\underset{i\in I^{+}}{\sum }\log\left(p_{i}\right)+\underset{i\in I^{-}}{\sum }\log\left(1-p_{i}\right),$$ (7)

where *L* is the log-likelihood function, *β* is the vector of parameters, which is estimated by maximizing the log likelihood, and *p*_*i*_ is the probability of the *i*-th example being in the rare class. To assign a class weight to each class, we modify *L*(*β*) as follows:

$$L\left(\beta \right)=w^{+}\underset{i\in I^{+}}{\sum }\log\left(p_{i}\right)+w^{-}\underset{i\in I^{-}}{\sum }\log\left(1-p_{i}\right),$$ (8)

where *w* ^+^ and *w* ^−^ are class weights assigned to the positive class and the negative class, respectively.

### 4.2 Weighted Random Forest

Random Forest uses decision trees as building blocks (Breiman, 2001). At each split, a predictor *x*_*j*_ and its corresponding cut point are chosen to minimize the misclassification error, which in practice is replaced by the Gini index (Hastie et al., 2009).

$$Giniindex=1-\overset{2}{\underset{i=1}{\sum }}{\left(\frac{n_{i}}{\overset{2}{\underset{j=1}{\sum }}n_{j}}\right)}^{2},$$ (9)

where *n*_*i*_ is the number of training observations of class *i* in the node under consideration.

To incorporate class weights into Random Forest, the weighted Gini index is given as follows:

$$WeightedGiniindex=1-\overset{2}{\underset{i=1}{\sum }}{\left(\frac{w_{i}n_{i}}{\overset{2}{\underset{j=1}{\sum }}w_{j}n_{j}}\right)}^{2},$$ (10)

where *w*_*i*_ is the weight for class *i*, and *i* ∈ {1, 2}.

### 4.3 Weighted Support Vector Machine

Support vector classifier is a maximum-margin classifier. The classification problem can be expressed as the following optimization problem (Hastie et al., 2009):

$$\min_{\beta_{0},\beta }\;\left\{w\overset{N}{\underset{i=1}{\sum }}\left(1-y_{i}f\left(x_{i}\right)\right)+\frac{1}{2}∥\beta {∥}^{2}\right\},$$ (11)

where *y*_*i*_ is the *i*-th observation, and *f*(*x*_*i*_) is the classification result for the *i*-th data point *x*_*i*_. The classifier *f*() is a function of parameters *β* and *β*_0_. Let *ϕ*(*x*_*i*_) be the transformed feature vector for *x*_*i*_, then *f*(*x*_*i*_) = *β* ^*T*^ *ϕ*(*x*_*i*_) + *β*_0_. Function *ϕ*() can be the identity function for linear classification problems. The parameters *β* and *β*_0_ are estimated by solving the optimization problem in (11). Since both *y*_*i*_ and *f*(*x*_*i*_) take values in {−1, +1}, *y*_*i*_ *f*(*x*_*i*_) = − 1 only when there is a classification error. Classification error is penalized by using the same weight *w* on both the positive class and the negative class. To incorporate class weights into SVM, we assign different weights to different classes as follows (Veropoulos et al., 1999; Batuwita et al., 2013):

$$\min_{\beta_{0},\beta }\;\left\{w^{+}\underset{i\in I^{+}}{\sum }\left(1-y_{i}f\left(x_{i}\right)\right)+w^{-}\underset{i\in I^{-}}{\sum }\left(1-y_{i}f\left(x_{i}\right)\right)+\frac{1}{2}∥\beta {∥}^{2}\right\},$$ (12)

where *w* ^+^ and *w* ^−^ are weights applied to the positive class and the negative class, respectively. Chang and Lin (2011) used a different formulation for the optimization problem, but the two formulations are equivalent. The equivalence of the two formulations can be found in Hastie et al. (2009).

## 5 Experiments

Through experiments on real datasets, we show the excellent performance of the proposed algorithms, and compare them with other algorithms: AdaBoost (Freund and Schapire, 1997), a simple weighting method that uses the ratio of positive cases and negative cases in the sample to compute weights, i.e., $\frac{w^{+}}{w^{-}}=\frac{N^{-}}{N^{+}}$ , and the unweighted method. We compare the algorithms in three aspects: 1) testing error, 2) the Receiver Operating Characteristic (ROC) curve, and 3) sensitivity to noise. All classifiers are given the same input. Finally, we compare the rare class performance of our algorithms with the group of algorithms that were developed from AdaBoost.

### 5.1 Testing Error

**FIGURE 2:**
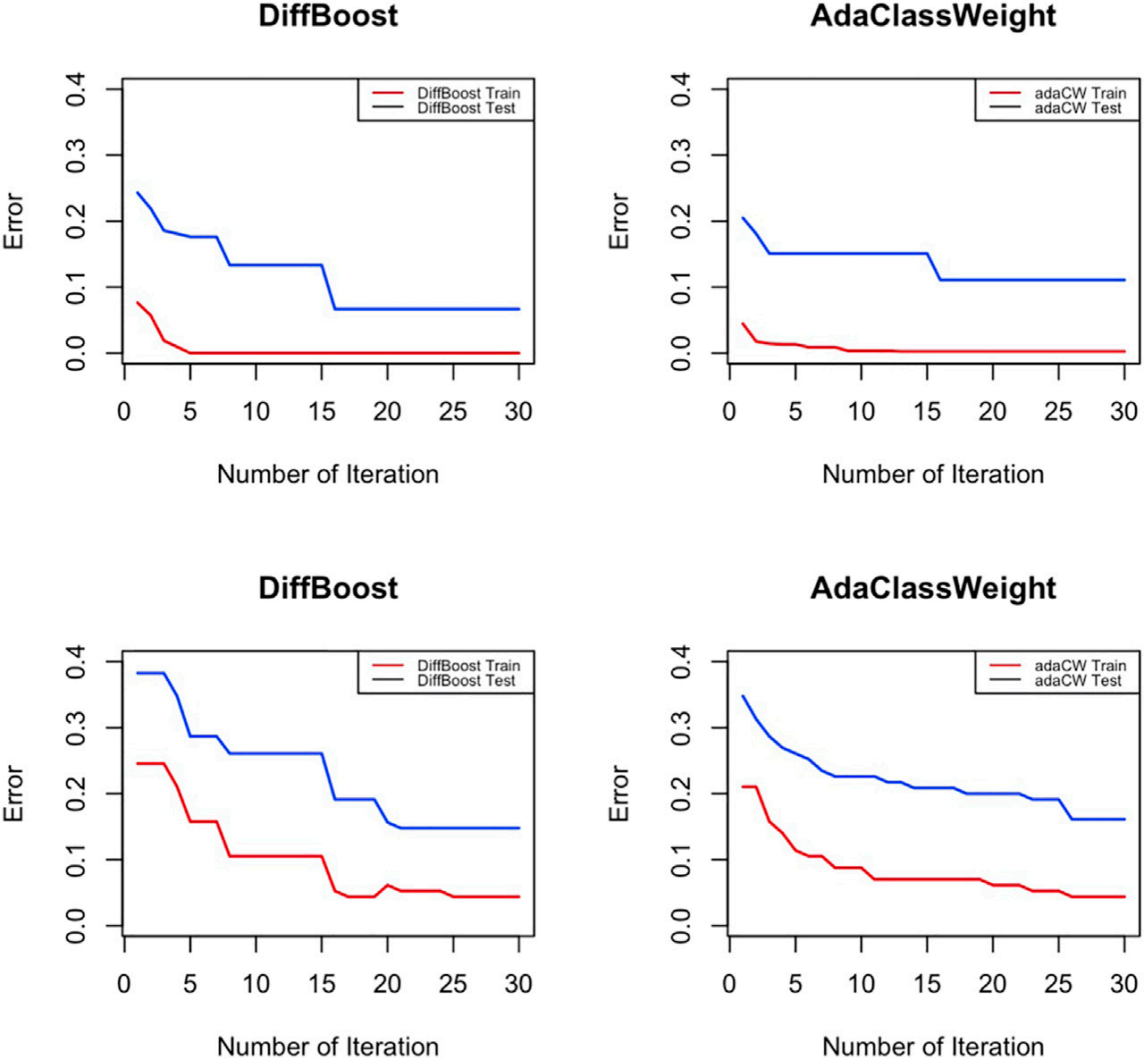
Testing error of the rare class decreases along with training error. In the top row, IEEE 39-bus power system data is used; In the bottom row, Spam data is used.

Training classifiers under differentiated weights significantly reduces the training error of the rare class. Will this also translate to a reduced error on the testing data? In this experiment we test if reducing training error on the rare class leads to overfitting, which means we will get an increased testing error. Figure 2 shows an overall trend of decreasing for both testing error and training error, so the improvement on training error on the rare class is also an improvement on the testing error. The algorithms start with an unweighted algorithm (*w* ^+^ = *w* ^−^ = 1 at initialization), and then through iterations as the training error decreases, the testing error also decreases. Results in Figure 2 are obtained from running Logistic Regression on the IEEE 39-bus dataset and the Spam dataset. Other classifiers show similar results.

Comparison with other methods on testing error are shown in Table 3. We consider three classifiers: LR, RF, and SVM. When we train a weak classifier under weights, LR can take both individual weights and class weights, but RF and SVM can only take class weights. Since AdaBoost produces one weight per example and does not give class weights, we can only obtain results for AdaBoost when using LR as the weak classifier. This experiment demonstrates that DiffBoost and AdaClassWeight both outperform existing methods in improving the rare class performance. The improvement for SVM is the most significant as the error rate is improved from 0.674 to 0.025.

**TABLE 3:** Testing error $\epsilon_{test}^{+}$ for the 39-bus power system data.

Methods	Testing error		
	LR	RF	SVM
AdaBoost	0.186	—	—
$\frac{w^{+}}{w^{-}}=\frac{N^{-}}{N^{+}}$	0.097	0.2	0.139
$\frac{w^{+}}{w^{-}}=1$	0.132	0.289	0.674
DiffBoost	0.067	0.089	0.025
AdaClassWeight	0.09	0.133	0.118

### 5.2 Receiver Operating Characteristic

It is reasonable to expect that the weighting algorithm will improve the prediction accuracy of the rare class at the expense of the main class. In this experiment, we will find out how much tradeoff exists between the rare class and the main class. We evaluate the performance of DiffBoost and AdaClassWeight in terms of the true positive rate and the false positive rate, and we compare them with 1) the simple weighting algorithm that uses the ratio to decide weight, i.e., $\frac{w^{+}}{w^{-}}=\frac{N^{-}}{N^{+}}$ , 2) the classical boosting algorithm AdaBoost, and 3) the unweighted algorithm (labeled as 1:1 in figures). AdaBoost is not designed to address the data imbalance problem, but it does use boosting to improve prediction performance without distinction of classes. We would like to see how differentiated boosting performs in terms of ROC compared to AdaBoost and others.

**FIGURE 3:**
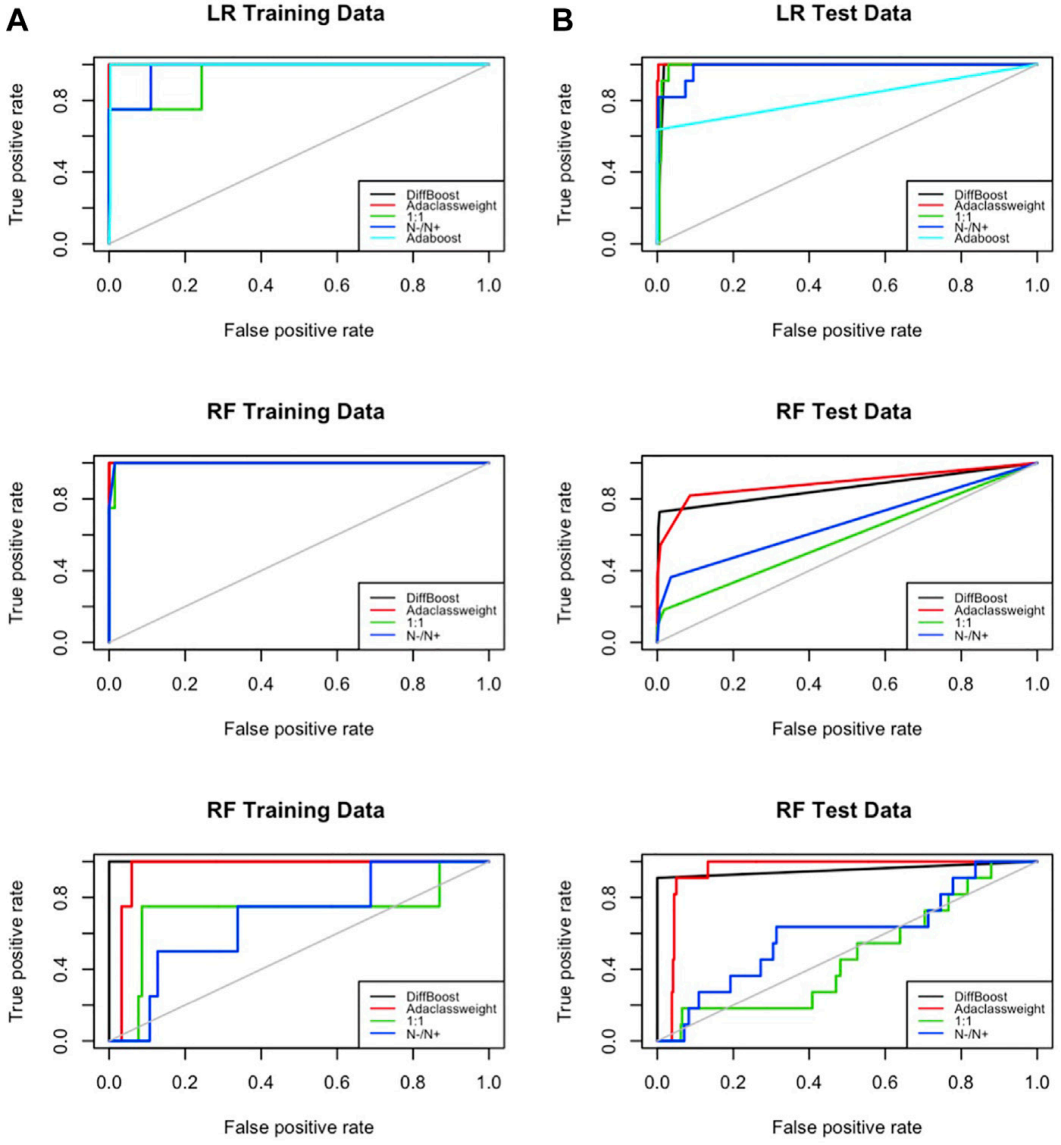
ROC on training data and testing data. Tests are done on the IEEE 39-bus power system data. DiffBoost and AdaClass Weight both have a larger area under the curve than other algorithms.

The ROC curve is the plot of true positive rate versus false positive rate for different cut-off points of a parameter. If increased true positive rate is at the cost of the increased false positive rate, the curve would go along the 45° line (the gray line in Figure 3). Otherwise, if the false positive rate does not go up proportionally, it would stay above the 45° line. On the ROC plot, the *area under the curve* is used to compare algorithms. The one with the largest area under the curve is considered the best.

Results from using IEEE 39-bus system data (Figure 3) show that the areas under the curves for the two proposed algorithms are the two largest compared to all other algorithms. This means the false positive rate did not go up proportionally. This is because there are a large number of examples in the main class, and adding appropriate weight on the rare class does not affect the performance of the main class too much.

### 5.3 Sensitivity to Noise

We care about sensitivity to noise because added weight will also amplify noise if noise happens to be on the rare class. In this section we test if the weighting algorithms DiffBoost and AdaClassWeight are sensitive to noise. We test the algorithms on the IEEE 39-bus dataset and present the results from using Logistic Regression. Results from using the other two classifiers are similar.

**FIGURE 4:**
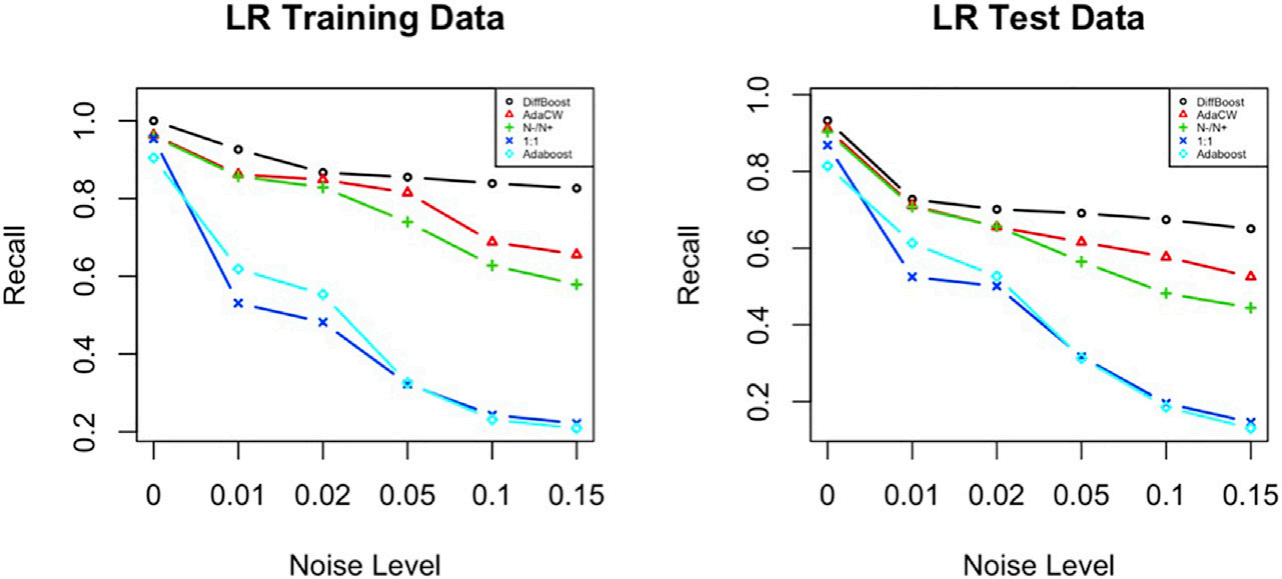
Sensitivity to noise for both training data and testing data. Test is done on the IEEE 39-bus power system data. While all algorithms are impacted by noise, the proposed algorithms perform better.

Noise is added into data as class noise, i.e., we flip a certain percentage of labels in each class. We define the percentage of flipped labels as the noise level, which takes values in (0, 1, 2, 5, 10, 15*%*). The results show that DiffBoost and AdaClassWeight can perform better than the ratio-based weighting algorithm, better than the AdaBoost algorithm, and much better than the unweighted algorithm (see Figure 4). The superiority of the two proposed algorithms is more evidenced as the noise level increases. Although the performance of DiffBoost and AdaClassWeight is also impacted by noise, the impact is smaller than other algorithms since the performance drop is not very steep.

### 5.4 Comparison With the Boosting Algorithms

The boosting algorithms AdaC1, AdaC2, AdaC3, and Adacost mentioned in Section 2 are developed from AdaBoost and all have similar ROC curves as AdaBoost (see Figure 3). Next we show another aspect of these algorithms: all methods require a preset cost *C*_*i*_, *∀i*, and the classification performance is extremely sensitive to this hyper-parameter. We use the Spam data to demonstrate. The Spam data has a total of 4,597 examples with 2,785 non-spam emails and 1,812 spam emails. We randomly select 1,506 examples from the original dataset. The ratio between non-spam examples and spam examples is 12 : 1, and 50–50*%* split is used for training and test within each class.

To see the effects of different cost settings on classifiers, we compare the results of Adacost, AdaC1, AdaC2, and AdaC3 based on different cost ratios *C*_+_: *C*_−_, and report the results on the test data (see Table 4). First of all, the highest recalls of the four algorithms occur at very different locations even for the same dataset. This indicates it is not a simple task to assign cost ratios. In Table 4 the highest recall is highlighted for each algorithm and the precision under the same cost ratio is also reported. Although these recalls are very impressive, the precisions are extremely low. If an algorithm simply predicts all cases as the rare class, it can achieve recall 1.0, but the precision is close to zero. This is observed with these boosting algorithms. In comparison, AdaClassWeight has the highest precision while achieving a high recall. It is also observed that the sum of recall and precision is the highest by the two proposed algorithms.

**TABLE 4:** Spam data. From left to right: the recall on test data under various cost ratios, the precision corresponding to the highest recall, training time, and test time.

	Recall with different cost ratios *C*_+_: *C*_−_							Precision	Training Time(s)	Test Time(s)
	1:1	1.5:1	2:1	2.5:1	5:1	7.5	10:1			
Adacost	0.633	0.669	0.698	0.878	0.698	0.92	0.775	0.1	1.41	0.004
AdaC1	0.633	0.824	0.885	0.896	0.917	0.99	0.99	0.076	3.51	0.004
AdaC2	0.633	0.9	0.92	0.96	0.98	0.99	0.99	0.076	2.73	0.005
AdaC3	0.594	0.881	0.94	0.824	0.824	0.775	0.91	0.33	2.1	0.004
DiffBoost	0.931	0.931	0.931	0.931	0.931	0.931	0.931	0.392	1.35	0.003
AdaClassWeight	0.92	0.92	0.92	0.92	0.92	0.92	0.92	0.403	0.74	0.003

The proposed algorithms also outperform the boosting algorithms in algorithm complexity. For the boosting algorithms, the cost ratio is a hyper-parameter. The optimal value for the hyper-parameter is obtained by a grid search. In the experiment, we performed a grid search for the cost ratio *C*_+_/*C*_−_ ∈ (Pazzani et al., 1994; King and Zeng, 2001) with step size 0.5. The training time would be much longer had we used a finer grid. In comparison, the proposed algorithms adaptively find the weights without a preset hyper-parameter. DiffBoost and AdaClassWeight terminate after *T* iterations, or before *T* iterations if a desired tradeoff between the positive class and the negative class have been found, so the algorithms are bounded to *T* iterations. The proposed algorithms have lower complexity as shown in Table 4.

## 6 Conclusion and Outlook

We have studied the problem of classifying rare events in imbalanced datasets, in which the rare class examples are significantly fewer than the main class examples. We focused on designing weighting algorithms to compute class weights during the training phase. DiffBoost and AdaClassWeight are general weighting algorithms and can be used in junction with any classifier. It has been tested with Logistic Regression, Random Forest, and Support Vector Machine, and has been applied to several datasets. The experimental results show that they improve the prediction accuracy of the rare class with a controlled tradeoff in the main class. The ROC curves of the proposed algorithms have larger “area under the curve” than the simple ratio-based algorithm, the original unweighted algorithm, and the AdaBoost algorithm as well as other boosting algorithms based on AdaBoost. It also has the advantage of being able to focus on the rare class, giving it a much higher accuracy in case we need to prioritize the rare class, with a controllable tradeoff. Multiclass classification for more than two classes using differential class weights is an easy extension from this work, which will be addressed in the future work.

## Data Availability

The datasets presented in this study can be found in online repositories. The names of the repository/repositories and accession number(s) can be found below: https://github.com/jhe58/Identification-of-Rare-Events-from-Imbalanced-Datasets.
